# Morphological Evolution of Vertically Standing Molybdenum Disulfide Nanosheets by Chemical Vapor Deposition

**DOI:** 10.3390/ma11040631

**Published:** 2018-04-20

**Authors:** Song Zhang, Jiajia Liu, Karla Hernandez Ruiz, Rong Tu, Meijun Yang, Qizhong Li, Ji Shi, Haiwen Li, Lianmeng Zhang, Takashi Goto

**Affiliations:** 1 State Key Laboratory of Advanced Technology for Materials Synthesis and Processing, Wuhan University of Technology, Wuhan 430070, China; jiajialiu@whut.edu.cn (J.L.); kaheruz30@gmail.com (K.H.R.); liyangmeijun@163.com (M.Y.); li.haiwen.305@m.kyushu-u.ac.jp (H.L.); lmzhang@whut.edu.cn (L.Z.); 2 Hubei Key Laboratory Advanced Technology of Automobile Parts, Wuhan University of Technology, Wuhan 430070, China; qizhongli@whut.edu.cn; 3 School of Materials and Chemical Technology, Tokyo Institute of Technology, Tokyo 152-8552, Japan; Shi.j.aa@m.titech.ac.jp; 4 International Research Center for Hydrogen Energy, Kyushu University, Fukuoka 819-0395, Japan; 5 Institute for Materials Research, Tohoku University, Sendai 980-8577, Japan; goto@imr.tohoku.ac.jp

**Keywords:** MoS_2_ nanosheets, chemical vapor deposition, hydrogen evolution reaction

## Abstract

In this study, we demonstrated the chemical vapor deposition (CVD) of vertically standing molybdenum disulfide (MoS_2_) nanosheets, with an unconventional combination of molybdenum hexacarbonyl (Mo(CO)_6_) and 1,2-ethanedithiol (C_2_H_6_S_2_) as the novel kind of Mo and S precursors respectively. The effect of the distance between the precursor’s outlet and substrates (denoted as *d*) on the growth characteristics of MoS_2_, including surface morphology and nanosheet structure, was investigated. Meanwhile, the relationship between the structure characteristics of MoS_2_ nanosheets and their catalytic performance for hydrogen evolution reaction (HER) was elucidated. The formation of vertically standing nanosheets was analyzed and verified by means of an extrusion growth model. The crystallinity, average length, and average depth between peak and valley (*R*z) of MoS_2_ nanosheets differed depending on the spatial location of the substrate. Good crystalized MoS_2_ nanosheets grown at *d* = 5.5 cm with the largest average length of 440 nm, and the highest *R*z of 162 nm contributed to a better HER performance, with a respective Tafel slope and exchange current density of 138.9 mV/decade, and 22.6 μA/cm^2^ for raw data (127.8 mV/decade and 19.3 μA/cm^2^ for iR-corrected data).

## 1. Introduction

Chemical vapor deposition (CVD) is considered to be the most practicable technique for synthesizing large-area, high quality, and size and thickness controllable MoS_2_ with different morphologies [1], including nanotubes [2], nanowires [3], nanosheets [4,5,6], etc., which are related to the anisotropic structure of MoS_2_. Vertically standing MoS_2_ nanosheets have aroused great interest from researchers due to their extensively exposed edge sites arising from a high-aspect-ratio nanostructure, which hold great potential for diverse applications such as hydrogen evolution reaction (HER) [7], hydrogen storage devices [8], lithium ion batteries [9], supercapacitors [10], and hydrodesulfurization catalysis [11], as well as for biological applications [12]. Both theoretical and experimental studies have indicated that the sulfide-terminated Mo-edge sites of MoS_2_ are catalytically active, while the basal plane remains inert [4,13,14,15,16,17]. Li found that larger, dense exposed edges of vertical MoS_2_ sheets can lead to a more active HER electro-catalyst [15]. However, while extensive studies have been devoted to two-dimensional materials which lie flat on the substrates [18,19,20,21], few reports have been developed regarding the growth of vertically standing MoS_2_ nanosheets [6,11,22,23,24,25,26]. The growth of such vertically standing nanosheets is still challengeable, and more efforts should be made to explore the growth characteristics of nanosheets. In previous studies, the distance between the precursor’s outlet and substrates was found to be an important factor in the growth of MX_2_ (M = Mo, W; X = S, Se, Te) [1,27,28]. Wang [21] proved that MoS_2_ domains showed a regular morphology transformation and a size change with the increase in distance between the precursor’s outlet and the substrate. Lin [29] found that domain size and surface coverage were varied dramatically by changing the distance between source and growth substrates. By optimizing the growth conditions, they grew a single-crystalline MoS_2_ flake, larger than 300 μm in size. Therefore, tuning the source–substrate distance is a practical method of controlling the morphology and size of MoS_2_ nanosheets.

As for the precursors of MoS_2_ synthesis, molybdenum hexacarbonyl (Mo(CO)_6_) is commonly used as an Mo precursor to the growth of molybdenum compounds [30,31,32], including MoS_2_, as the melting point of Mo(CO)_6_ is quite low, meaning only a low energy is required to evaporate it, and making it easily controllable when adding it to the reaction chamber. 1,2-ethanedithiol (SHCH_2_CH_2_SH, or C_2_H_6_S_2_), which remains in a liquid state at room temperature, is different to hydrogen sulfide (H_2_S) [30,31], and much less toxic than dimethyl disulfide (CH_3_SSCH_3_) [32,33,34], was adopted as an S precursor.

In this contribution, we provide a facile CVD method for depositing vertically standing MoS_2_ nanosheets on diverse substrates, using Mo(CO)_6_ and 1,2-ethanedithiol (C_2_H_6_S_2_) as the unconventional combination of precursors. MoS_2_ nanosheets were grown at different substrate spatial locations (i.e., the distance between the precursor’s outlet and the substrates, denoted as *d*). The effect of *d* on growth characteristics, and its relationship with catalytic performance were discussed.

## 2. Materials and Methods

**Figure 1:**
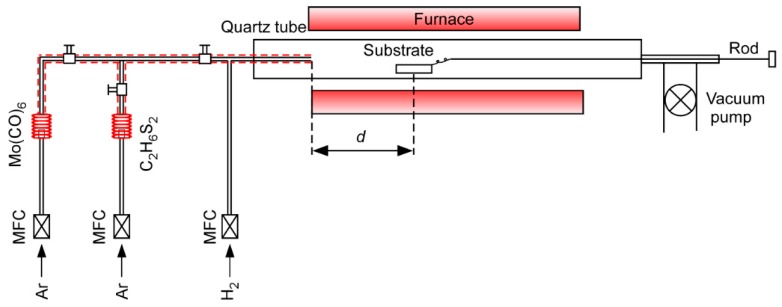
Schematic illustration of chemical vapor deposition (CVD) system.

Molybdenum hexacarbonyl (Mo(CO)_6_, 98.0%, Alfa Aesar, Shanghai, China) and 1,2-ethanedithiol (C_2_H_6_S_2_, 99.0%, TCI, Shanghai, China) were used as precursors for the growth of MoS_2_ nanosheets. The experimental setup is illustrated in Figure 1. The substrates, fused quartz (10 × 10 × 0.5 mm^3^), were cleaned successively in acetone, isopropyl alcohol (IPA), and deionized (DI) water in an ultrasonic bath for 10 min, and then dried with an ultra high purity (UHP) N_2_ stream. Mo(CO)_6_ and C_2_H_6_S_2_ were placed in vaporizers and maintained at 50 and 70 °C respectively. The vapor transport pipes were heated and kept at 80 °C. Ar and H_2_ were used as carrier gas and reducing gas respectively. The substrate was placed on an alumina ceramic boat in the setting position of the tube, and the distance between the precursor’s outlet and the substrates was denoted as *d* (as shown in Figure 1). The furnace was heated at a rate of 10 °C/min to 700 °C under a H_2_ flow of 100 sccm. Following this, the H_2_ flow rate was set to 500 sccm, while the total pressure (*P*_tot_) reached 250 Pa. 40 sccm of Ar gas was allowed through vaporizers to carry both Mo(CO)_6_ and C_2_H_6_S_2_ vapors respectively. Mo(CO)_6_ and C_2_H_6_S_2_ precursors were introduced into the reactor at 700 °C for 5 min. After deposition, the furnace was naturally cooled down to room temperature by a H_2_ flow of 100 sccm at *P*_tot_ = 100 Pa.

X-ray diffraction microscopy (XRD; Ultima III, Rigaku, Tokyo, Japan, at 40 kV and 40 mA), Raman spectroscopy (Labram HR Evolution, Horiba, Kyoto, Japan, with a 532 nm laser) and Transmission electron microscopy (TEM; JEOLJEM-2100, Tokyo, Japan, at 200 kV) were used to characterize the microstructure of vertically standing MoS_2_ nanosheets. A Field-emission scanning electron microscope (FESEM; Quanta-250, FEI, Houston, TX, USA, at 20 kV) and Atomic force microscopy (AFM; Vecco Nanoscope IIIa, Veeco, Plainview, NY, USA, tapping mode) were used to observe the surface morphology of vertically standing MoS_2_ nanosheets, and the average depth between peak and valley (*R*z) of the nanosheets was determined from the AFM height profile. X-ray photoelectron spectroscopy (XPS; ESCALAB 250Xi, Thermo Fisher Scientific, Waltham, MA, USA, electron spectrometer using Al *K*α-radiation) was conducted to determine the components and binding energies of MoS_2_ film. Nano measurer software (Nano measurer 1.2.5, Jie Xu, Fudan University, Shanghai, China) was used to measure and calculate the average length and area density of the nanosheets.

HER tests were carried out to evaluate the catalytic performance efficiency of the as-prepared vertically standing MoS_2_ nanosheets. All of the electrochemical measurements were performed in a 0.5 M H_2_SO_4_ solution by using a three-electrode setup on an electrochemical workstation (CHI660A, CH Instruments Inc., Austin, TX, USA), with a saturated calomel electrode (SCE) as the reference electrode, a graphite rod as the counter electrode, and vertically standing MoS_2_ nanosheets grown on an Au substrate as working electrodes. The reference electrode was calibrated with respect to a reversible hydrogen electrode (RHE). To perform the calibration, a platinum wire was used as the working electrode to run the cyclic voltammetry (CV) at a scan rate of 1 mV/s, and the average of the two potentials at which the current is zero was considered as the thermodynamic potential. Herein, E(RHE) = E(SCE) + 0.274 V.

## 3. Results

**Figure 2:**
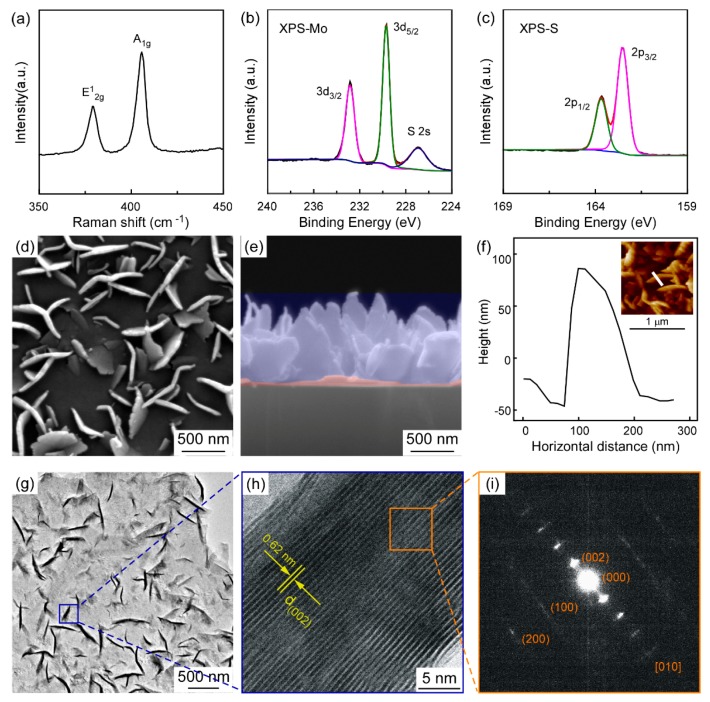
Characterizations of as-synthesized MoS_2_ nanosheets at *d* = 5.5 cm (distance between the precursor’s outlet and substrates, denoted as *d*) (**a**) Raman spectra; (**b**) XPS (X-ray photoelectron spectroscopy) of Mo 3*d* and S 2*s* peaks; (**c**) XPS spectra of S 2*p* peak; (**d**) top view; and (**e**) cross-section FESEM (Field-emission scanning electron microscope) images of vertically standing MoS_2_; (**f**) AFM (Atomic force microscopy) height profile of single MoS_2_ nanosheet, the insert shows the corresponding AFM image; (**g**) low-magnification and (**h**) high-magnification TEM (Transmission electron microscopy) images of MoS_2_ nanosheets; (**i**) the corresponding selected area electron diffraction pattern.

Firstly, characterizations of a typical MoS_2_ nanosheet deposited at *d* = 5.5 cm are displayed in Figure 2. Two Raman characteristic bands at 406 and 379 cm^−1^ with full-width half-maximum (FWHM) values of 5.5 and 5.6 cm^−1^ are exhibited in Figure 2a, corresponding to the A_1g_ and E^1^_2g_ modes of hexagonal MoS_2_ respectively [33,34,35]. A_1g_ and E^1^_2g_ vibrational modes are associated with the out-of-plane vibration of sulfur atoms, and the in-plane vibration of Mo and S atoms [33,34,35]. The E^1^_2g_/A_1g_ ratio of vertically standing MoS_2_ nanosheets is 0.42, while that of single-crystal bulk MoS_2_ obtained by mechanical exfoliation is 0.71 [6]. Based on this fact, the E^1^_2g_/A_1g_ relative ratio calculated from the Raman results can be used to distinguish between basal- (>0.5) and edge-oriented (<0.5) MoS_2_ [36,37,38]. Consequently, it can be inferred that MoS_2_ grown at *d* = 5.5 was revealed to be edge-oriented, which means there are more exposed edges in vertically standing MoS_2_ films than in single-crystal bulk MoS_2_.

Figure 2b,c shows the XPS spectra of as-grown MoS_2_ film for Mo 3*d* and S 2*p* binding energies respectively. Mo 3*d* spectra peaks [39] at two binding energies of 229.87 and 223.07 eV, corresponding to 3*d*_5/2_ and 3*d*_3/2_ of Mo^4+^. S 2*p* [40,41] exhibits two characteristic peak positions at binding energies of 162.7 eV and 163.8 eV, which are attributed to spin–orbit 2*p*_3/2_ and 2*p*_1/2_ of S^2−^ respectively. Top view and cross-section FESEM images of vertically standing MoS_2_ are presented in Figure 2d,e. MoS_2_ films consist of two parts (see Figure 2e): The part marked with red shadow is determined to be basal-oriented MoS_2_ film, while the upper part marked in blue is nanosheets standing upright, which proves that the MoS_2_ nanosheets were vertically grown on the substrate. Figure 2f shows the AFM height profile of a single MoS_2_ nanosheet, indicating that the height of the single sheet is about 150 nm (from peak to valley).

TEM measurements were carried out in order to further characterize the crystal structure of the vertically standing MoS_2_ nanosheets. A sample was prepared by scraping the MoS_2_ nanosheets from the substrate, after which it was dissolved in alcohol, and the solution was dropped on a TEM grid. Figure 2g shows the low-magnification image of MoS_2_ nanosheets, containing plentiful vertically standing nanosheets (darker part). Figure 2h records the high-magnification TEM image of the square marked in Figure 2g, which is the vertically standing MoS_2_ nanosheets. As shown in Figure 2h, a layered structure of MoS_2_ sheets, with a distance of 0.062 nm between two MoS_2_ layers was obtained, suggesting that the crystal’s orientation is (002) direction [7,24,38]. It can be determined that the stacking number of MoS_2_ nanosheets is 46-layer (Figure 2h), while the stacking number of nanosheets presented in Figure 2g is in the range of 6‒50 layers. Figure 2i shows the corresponding selected area electron diffraction (SAED) pattern of the square marked in Figure 2h. Regularly arranged diffraction spots are indexed as a crystal face of (002) and (100) respectively, indicating a [010] zone axis and a hexagonal crystal structure of 2H-MoS_2_.

Currently, out of the extensive growth mechanisms for vertically standing MoS_2_ nanosheets proposed in the literature, the most acceptable mechanism is the so-called extrusion growth model, which demonstrates that the growth of edge-oriented MoS_2_ nanosheets occurs after the formation of a basal-oriented structure [6]. This growth model speculates that the basal-oriented multiple MoS_2_ layers are separately formed on the substrate surface, after which these layers reach a critical thickness, and form thick island-shaped films. Given increasing growth time, isolated thick films will become large enough to connect with each other; at this point, the edge of these island-shaped MoS_2_ layers tend to stand up due to the extrusion between adjacent basal-oriented planes, thus producing edge-oriented MoS_2_ nanosheets.

Based on our experiment results, a thin flat layer can be clearly observed from a cross-section image (Figure 2e), which corresponds to the basal-oriented MoS_2_ layer. Furthermore, a few of the MoS_2_ slabs were not vertically aligned, but rather tilted on the base surface (see Figure 2d), which could be ascribed to the asymmetrical extrusion effect. Briefly, the observation of these basal-oriented layers and tilted slabs offers direct evidence of the formation of vertically standing MoS_2_ nanosheets, fully consistent with the proposed extrusion growth model.

**Figure 3:**
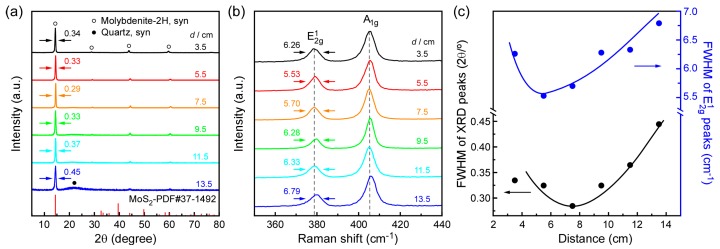
(**a**) XRD spectra and (**c**) the corresponding FWHM of (002) peak, (**b**) Raman spectra (**c**) the corresponding FWHM of E^1^_2g_ peak.

The XRD and Raman results of MoS_2_ nanosheets are presented in Figure 3. A strong sharp (002) diffraction peak is observed at 2*θ* = 14.5°, and higher order peaks (004), (006), and (008) of MoS_2_ have also appeared. All of these peaks can be indexed as the pure hexagonal MoS_2_ phase with lattice constants of *a* = 3.161 Å and *c* = 12.299 Å (PDF No. 37-1942), as no diffraction peaks from impurities are observed in the XRD pattern. Figure 3c depicts the variation in FWHM value of the (002) diffraction peak. As *d* increases, the FWHM value of this peak decreases at first, and then increases, showing a minimum value of FWHM at *d* = 7.5 cm, implying a better crystallinity in (002) direction. Two distinctive Raman peaks, E^1^_2g_ (~379 cm^−1^) and A_1g_ (~405 cm^−1^), of MoS_2_ are presented in Figure 3b. The FWHM value of E^1^_2g_ peaks can be used as an indicator for MoS_2_ crystalline quality [42], which is summarized in Figure 3c, and exhibits the same tendency as the value of XRD peaks, showing however a lowest value at *d* = 5.5 cm. This inconsistency between XRD and Raman measurements may be attributable to the different sources of FWHM value. Furthermore, the E^1^_2g_/A_1g_ ratios of vertically standing MoS_2_ nanosheets at *d* from 3.5 to 13.5 cm are all less than 0.5, and the values are 0.41, 0.42, 0.42, 0.41, 0.39, 0.40 respectively, indicating that deposited MoS_2_ layers are edge-oriented. Additionally, the gap between E^1^_2g_ and A_1g_ modes ranges from 25.3 to 26.9 cm^−1^, indicating that the stacking number of MoS_2_ is 6-layer or more, which refers to a bulk character instead of a thin, few-layered 2D film [32]. This result was consistent with the TEM observation in Figure 2g.

**Figure 4:**
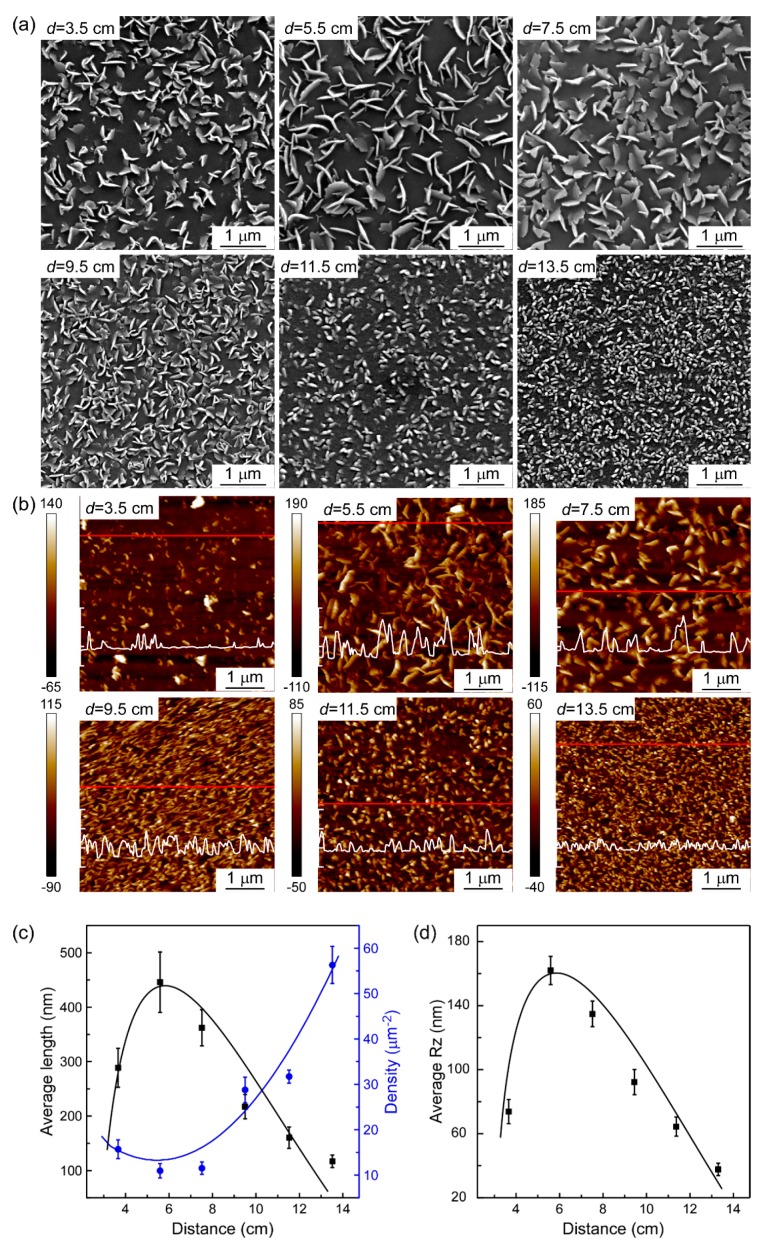
FESEM (**a**) and AFM (**b**) images of MoS_2_ nanosheets deposited at different *d* values (3.5, 5.5, 7.5, 9.5, 11.5, 13.5 cm). The white lines overlapped on the AFM images represent the height profiles for the red lines, the scale bar is 100 nm for each interval; (**c**) Effect of *d* on the average length and area density (which are calculated from measured statistics of single sheet lengths and numbers by Nano measurer software) of the nanosheets. (**d**) Effect of *d* on average depth between peak and valley (*R*z).

The growth characteristics of MoS_2_ deposited at different spatial locations are summarized in Figure 4. MoS_2_ nanosheets did not lay flat on the surface of the substrate, but rather appeared to stand erect with different angles, undergoing drastic morphological changes in shape, size, density, and texture. Firstly, from the FESEM and AFM images shown in Figure 4a,b, with the increasing *d*, the morphology of MoS_2_ changed from vertically standing nanosheets (*d* ≤ 9.5 cm) into nanoparticles (*d* ≥ 11.5 cm). Secondly, the average length of MoS_2_ nanosheets calculated by Nano measurer software first increases, and then decreases. MoS_2_ grown at *d* = 5.5 cm indicated the largest average length, which can reach a maximum of up to 440 nm. In comparison, the area density of MoS_2_ showed the lowest value where the MoS_2_ sheets had the largest average length. Thirdly, although the MoS_2_ prepared at *d* in the range of 5.5–9.5 stood vertically on the quartz substrate, nanosheets with three different textures, i.e., three-petal-shaped (*d* = 5.5 cm), leaf-shaped (*d* = 7.5 cm), and triangle-shaped (*d* = 9.5 cm), were observed respectively. The average depth between peak and valley (*R*z) is closely related to the MoS_2_ nanostructure, and can be explained by the average height of the nanosheets. The *R*z value of MoS_2_ shows the highest value at *d* = 5.5 cm, leading to a maximum of 162 nm. Based on the above results, it can be concluded that sparsely distributed MoS_2_ with a lower area density is beneficial to the growth of large size MoS_2_ nanosheets, and contributes to a higher vertically standing structure.

**Figure 5:**
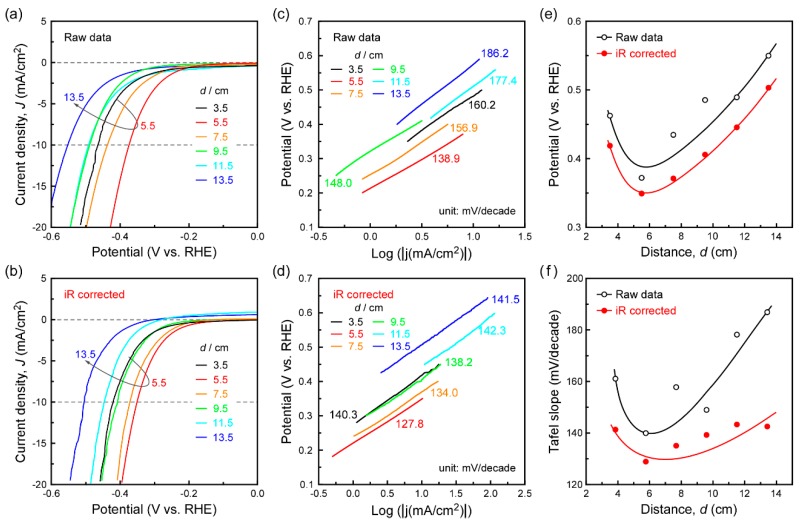
Electrochemical characterization of vertically standing MoS_2_ nanosheets grown on Au foils at different *d*. Polarization curves of raw data (**a**) and iR-corrected data (**b**); corresponding Tafel plots of raw data (**c**) and iR-corrected data (**d**); potential value to achieve −10 mA/cm^2^ (**e**) and Tafel slope (**f**) at different *d*.

A typical three-electrode device for HER test was carried out to evaluate the catalytic activity. Vertically standing MoS_2_ nanosheets were grown on Au substrates by using the same procedure as previously described on the quartz substrate. Figure 5a–d demonstrates the typical cathodic polarization curves and corresponding Tafel plots ((a,c) for the raw data; (b,d) for the iR-corrected data). The potential for critical current density (*J*) of 10 mA/cm^2^ is considered to be a common feature of merit to evaluate the efficiency of the HER catalyst [25,43]. In Figure 5e, potential for 10 mA/cm^2^ shows a minimum value at *d* = 5.5 cm, indicating a good catalytic performance. An overpotential of 371 mV is needed to achieve 10 mA/cm^2^, while correcting the raw data for iR losses revealed an even more impressive performance (a lower overpotential of 348 mV). The Tafel slope is an inherent property of the catalyst, which is determined by the rate-limiting step of HER [7,8,44]. Figure 5f shows the Tafel slope of MoS_2_ grown at different *d*: the Tafel slope is 138.9 mV/decade for raw data, and 127.8 mV/decade for iR-corrected data at *d* = 5.5 cm. The above Tafel slope value lies in the medium region compared to previous studies, which show a wide range of Tafel slopes from 40 mV to 212 mV/decade [25,45,46,47]. The exchange current density *j*_0_ was determined by fitting the linear portion of the Tafel plot at a low cathodic current of the Tafel equation [15]. Based on the above results, it can be found that the most appropriate position for the growth of MoS_2_ nanosheets was *d* = 5.5 cm, showing the highest HER performance with the exchange current density *j*_0_ of 22.6 μA/cm^2^ (19.3 μA/cm^2^ for iR-corrected data)—among the medium values reported for MoS_2_ catalysts (0.025−38.9 μA/cm^2^) [12,48,49]. Combined with the growth characteristics and HER performance results discussed above, it could be concluded that MoS_2_ nanosheets with a larger average length, and higher *R*z contributed to a better HER performance, ascribed to more exposed edge-sites in such vertically standing nanostructures.

## 4. Conclusions

Vertically standing MoS_2_ nanosheets were synthesized by the CVD method with an unconventional combination of molybdenum hexacarbonyl (Mo(CO)_6_) and 1,2-ethanedithiol (C_2_H_6_S_2_) precursors. Spatial location (i.e., the distance between precursor’s outlet and the substrates, denoted as *d*) played an important role in the growth characteristics of MoS_2_. XRD patterns and Raman indicated that 2H-MoS_2_ with good crystallinity in (002) direction was obtained, and the FWHM value of (002) diffraction and E^1^_2g_ peaks reached the minimum value at *d* = 5.5 cm, implying the highest crystallinity. Similarly, MoS_2_ nanosheets grown at this location possessed the largest average length, highest *R*z, and lowest area density. The vertically standing structure of edge-oriented MoS_2_ originated from the extrusion between adjacent basal planes. Electrochemical characterization displayed that MoS_2_ nanosheets with a larger average length and a higher *R*z contributed to a better HER performance. To conclude, the most appropriate substrate spatial location is considered to be *d* = 5.5 cm, where the as-deposited vertically standing MoS_2_ nanosheets exhibited the largest average length of 440 nm, and the highest *R*z of 162 nm. This contributed to a better HER performance, leading to a respective Tafel slope and exchange current density of 138.9 mV/decade and 22.6 μA/cm^2^ for raw data (127.8 mV/decade and 19.3 μA/cm^2^ for iR-corrected data).
